# Correction: Mindfulness-Based Cognitive Therapy for Treatment-Resistant Depression: A protocol for systematic review and meta-analysis

**DOI:** 10.1371/journal.pone.0316594

**Published:** 2024-12-26

**Authors:** Michele F. Rodrigues, Larissa Junkes, Jose Appolinario, Antonio E. Nardi

The initials of the first and fourth authors are incorrectly indexed in PubMed. The correct initials are as follows: Rodrigues MF and Nardi AE.

The correct citation is: Rodrigues MF, Junkes L, Appolinario J, Nardi AE (2024) Mindfulness-Based Cognitive Therapy for Treatment-Resistant Depression: A protocol for systematic review and meta-analysis. PLoS ONE 19(10): e0306227. https://doi.org/10.1371/journal.pone.0306227.
