# Supplementary material for: Mindfulness-Based Cognitive Therapy for Treatment-Resistant Depression: A protocol for systematic review and meta-analysis
Source: PLoS One. 2024 Oct 15;19(10):e0306227. doi: 10.1371/journal.pone.0306227 (PMC11478908; doi:10.1371/journal.pone.0306227)
Supplement: S1 File — (DOCX) [file pone.0306227.s002.docx]

| **Search Strategies** | |
| --- | --- |
| **Databases** | **Search strategies** |
| **MEDLINE** | ((“Depressive Disorder, Major”;[Mesh] OR (Major Depressive Disorder) OR (Paraphrenia, Involutional) OR Involutional Paraphrenia*) OR (Paraphrenias, Involutional) OR (Psychosis, Involutional) OR (Involutional Psychos*) OR (Psychoses, Involutional) OR (Depression, Involutional) OR (Involutional Depression) OR (Melancholia, Involutional) OR (Involutional Melancholia)) OR (“Depressive Disorder, Treatment-Resistant”[Mesh] OR (Depressive Disorder*, Treatment Resistant) OR (Disorder*, Treatment-Resistant Depressive) OR (Treatment-Resistant Depressive Disorder*) OR (Refractory Depression*) OR (Depression*, Refractory) OR (Therapy-Resistant Depression) OR (Depression*, Therapy-Resistant) OR (Therapy Resistant Depression) OR (Therapy-Resistant Depressions) OR (Treatment Resistant Depression*) OR (Depression*, Treatment Resistant) OR (Resistant Depression*, Treatment))) AND (“Mindfulness”[Mesh]). |
| **PsycINFO** | "Major Depression" OR "Treatment Resistant Depression" AND "Mindfulness" |
| **Web of Science** | "Treatment-Resistant Depressive Disorder" OR "Refractory Depression" OR "Therapy-Resistant Depression" OR "Treatment Resistant Depression" AND "Mindfulness" |
| **ClinicalTrials.gov** | “Major Depression”; OR “Major Depressive Disorder”; OR “Involutional  Depression”; OR “Involutional Melancholia”; OR “Treatment-Resistant  Depression”; OR “;Refractory Depression”; OR “Therapy-Resistant Depression”;  AND “Mindfulness” |
| **EMBASE** | 'major depressive disorder' OR 'major depressive disorder' OR 'paraphrenia, involutional' OR 'involutional paraphrenia' OR 'paraphrenias, involutional' OR 'psychosis, involutional' OR 'involutional psychosis*' OR 'psychoses, involutional' OR 'depression, involutional' OR 'involutional depression' OR 'melancholia, involutional' OR 'involutional melancholia' OR 'treatment resistant depression'/exp OR 'depressive disorder, treatment resistant' OR 'disorder, treatment-resistant depressive' OR 'treatment-resistant depressive disorder*' OR 'refractory depression' OR 'depression*, refractory' OR 'therapy-resistant depression' OR 'depression, therapy-resistant' OR 'therapy resistant depression' OR 'therapy-resistant depressions' OR 'treatment resistant depression' OR 'depression, treatment resistant' OR 'resistant depression, treatment' AND 'mindfulness' |
